# Relationship of continuous glucose monitoring-related metrics with HbA1c and residual β-cell function in Japanese patients with type 1 diabetes

**DOI:** 10.1038/s41598-021-83599-x

**Published:** 2021-02-17

**Authors:** Naru Babaya, Shinsuke Noso, Yoshihisa Hiromine, Yasunori Taketomo, Fumimaru Niwano, Sawa Yoshida, Sara Yasutake, Yumiko Kawabata, Hiroshi Ikegami

**Affiliations:** Department of Endocrinology, Metabolism and Diabetes, Kindai University Faculty of Medicine, 377-2 Ohno-higashi, Osaka-sayama, Osaka, 589-8511 Japan

**Keywords:** Type 1 diabetes, Biomarkers

## Abstract

The targets for continuous glucose monitoring (CGM)-derived metrics were recently set; however, studies on CGM data over a long period with stable glycemic control are limited. We analyzed 194,279 CGM values obtained from 19 adult Japanese patients with type 1 diabetes. CGM data obtained during stable glycemic control over four months were analyzed. CGM-related metrics of different durations “within 120, 90, 60, 30, and 7 days” were calculated from baseline. Time in range (TIR; glucose 70–180 mg/dL), time above range (TAR; glucose ≥ 181 mg/dL), and average glucose levels, but not time below range (TBR; glucose ≤ 69 mg/dL), strongly correlated with glycated hemoglobin (HbA1c) values (P < 0.0001). TBR correlated with glucose coefficient of variation (CV) (P < 0.01). Fasting serum C-peptide levels negatively correlated with glucose CV (P < 0.01). HbA1c of approximately 7% corresponded to TIR of 74% and TAR of 20%. The shorter the CGM period, the weaker was the relationship between HbA1c and CGM-related metrics. TIR, TAR, and average glucose levels accurately reflected HbA1c values in Japanese patients with type 1 diabetes with stable glycemic control. Glucose CV and TBR complemented the limitation of HbA1c to detect glucose variability and hypoglycemia. Stable glycemic control with minimal hypoglycemia depended on residual β-cell function.

## Introduction

Glycated hemoglobin (HbA1c) is the gold standard for long-term blood glucose monitoring reflecting the mean glucose levels over the last 2–3 months^1,2^. Many seminal trials have clarified the relationship between glycemic control and microvascular and macrovascular complications in patients with type 1 and type 2 diabetes using HbA1c^3–5^. However, HbA1c has several limitations because first, it is affected by genetic, hematological, and other clinical factors unrelated to glycemia, such as disorders affecting red cell turnover, hemoglobinopathies, and certain anemias etc. Second, HbA1c does not reflect the short-term changes in glycemic control. Third, HbA1c cannot be used to identify the magnitude and frequency of intra- and inter-day glucose variations^1,2^ to distinguish between patients with stable glycemic control and those with rapidly changing glycemic levels.

Continuous glucose monitoring (CGM) has been recognized as a "beyond HbA1c" tool^1,6^. CGM devices, which can measure glucose levels in the subcutaneous interstitial fluid and convert them into the equivalent venous blood glucose levels, have grown rapidly in recent years, because of their improved sensor accuracy, convenience of use, and reduced user cost. The major advantage of CGM technology is that the overall daily profile of blood glucose can be captured, particularly, the postprandial and nocturnal blood glucose levels. Many studies have demonstrated considerable benefits of CGM in patients with diabetes^7^, particularly those with insulin-depended diabetes and at high hypoglycemia risk^2,8^. Although the opportunities to use CGM are increasing, the utilization of CGM data is not adequate in routine clinical practice because of the lack of clear and agreed-upon glycemic targets.

Recently, consensus recommendations for the relevant aspects of CGM data utilization have been reported by the consensus panel in the Advanced Technologies & Treatments for Diabetes (ATTD) Congress in 2019^2^. In the consensus recommendations, several metrics, such as time in range (TIR), time above range (TAR), time below range (TBR), average glucose, glycemic variability (% coefficient of variation [CV]), were included. Given that long-term studies on the relationship between these metrics and diabetes complications are required, the guidance on target for these metrics has been shown for various types of diabetes based on several lines of evidence, which showed correlations between TIR (70–180 mg/dL) and diabetes complications^7,9,10^ and clarified the relationship between TIR and HbA1c^11–13^. However, most studies were based on data extracted from published trials or articles for other purposes. In addition, these studies were performed in Western countries and most of the participants were Caucasian^11,13^. Considering the impact of the racial factor in the relationship between mean blood glucose and HbA1c^14^, studies in different populations are necessary.

This study aimed to clarify the relationship between several metrics measured by CGM and data of well-established laboratory parameters, particularly, HbA1c and serum C-peptide (C-peptide immunoreactivity: CPR), in adult Japanese patients with type 1 diabetes. To ensure data accuracy, CGM data for a sufficiently long period were obtained from patients with stable glycemic control without conditions leading to variability of HbA1c. This would be beneficial for both, patients with type 1 diabetes and healthcare professionals for the appropriate interpretation of CGM-related metrics and their use for better management of glycemic control, reducing the incidences of hypoglycemia or hyperglycemia.

## Results

**Figure 1:**
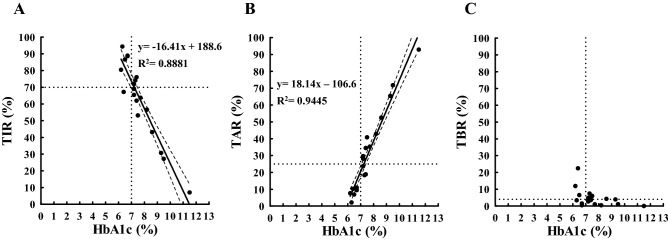
Scatterplot indicating the relationship between HbA1c (x-axis) and CGM-related metrics (TIR (**A**), TAR (**B**), and TBR (**C**); y-axis) measured within 120 days from baseline. The solid line is the line of best-fit from the linear regression analysis. The dashed band is the 95% confidence band of the best-fit line. The dotted horizontal lines represent a TIR of 70% (**A**), a TAR of 25% (**B**), and a TBR of 4% (**C**): these values are based on the targets of TIR, TAR, and TBR for adults with type 1 or type 2 diabetes from recommendations of the ATTD 2019 consensus statement. The dotted vertical lines represent an HbA1c of 7%. *TIR* time in range (glucose 70–180 mg/dL), *TAR* time above range (glucose ≥ 181 mg/dL), *TBR* time below range (glucose ≤ 69 mg/dL), *R*^*2*^ coefficient of determination.

The results of univariate regression analysis between HbA1c (x-axis) and TIR (y-axis) measured within 120 days from baseline are shown in Fig. 1A. A strong inverse relationship between HbA1c and TIR was observed (P < 0.0001), suggesting that HbA1c data were sufficient to explain TIR. HbA1c of 7% corresponded to a TIR of approximately 74% (Table 1), and the coordinate (x = 7%, y = 70%) was included within the 95% confidence band of the best-fit line (Fig. 1A). An increase in TIR of 18% will cause a decrease in HbA1c of 1.0% (Supplementary Fig. 1A). In Fig. 1B, a strong positive relationship was observed between HbA1c and TAR (P < 0.0001), suggesting that HbA1c data were sufficient to explain TAR. HbA1c of 7% corresponded to a TAR of approximately 20% (Table 1), and the coordinate (x = 7%, y = 25%) was not included within the 95% confidence band of the best-fit line (Fig. 1B). A decrease of 19% in TAR will cause a decrease in HbA1c of 1.0% (Supplementary Fig. 1B). In Fig. 1C, unlike TIR and TAR, no significant relationship was observed between HbA1c and TBR (P > 0.05) (Table 1).

**Table 1:** Linear regression showing the difference in the measurement periods.

Measurement period from baseline	y = TIR (%) or TAR (%) or TBR (%) or average glucose (mg/dL) x = HbA1c (%)				
	Best-fit line	95% CI of a slope	R^2^	P value	Value when x = 7.0
**TIR**					
Within 120 days	y = − 16.41x + 188.6	− 19.39 to − 13.43	0.8881	< 0.0001	73.73
Within 90 days	y = − 16.06x + 185.6	− 19.13 to − 12.98	0.8771	< 0.0001	73.18
Within 60 days	y = − 16.38x + 188.3	− 19.40 to − 13.36	0.8851	< 0.0001	73.64
Within 30 days	y = − 16.69x + 191.4	− 20.30 to − 13.09	0.8489	< 0.0001	74.57
Within 7 days	y = − 16.16x + 187.9	− 21.44 to − 10.89	0.7110	< 0.0001	74.78
**TAR**					
Within 120 days	y = 18.14x − 106.6	15.91 to 20.37	0.9445	< 0.0001	20.38
Within 90 days	y = 17.90x − 104.7	15.69 to 20.11	0.9449	< 0.0001	20.60
Within 60 days	y = 18.30x − 108.2	16.25 to 20.36	0.9541	< 0.0001	19.90
Within 30 days	y = 18.55x − 111.5	15.40 to 21.71	0.9005	< 0.0001	18.35
Within 7 days	y = 18.43x − 110.5	14.17 to 22.70	0.8302	< 0.0001	18.51
**TBR**					
Within 120 days	y = − 1.731x + 17.98	− 3.526 to 0.06383	0.1959	0.0578	5.863
Within 90 days	y = − 1.839x + 19.10	− 3.890 to 0.2112	0.1740	0.0756	6.227
Within 60 days	y = − 1.917x + 19.89	− 3.949 to 0.1137	0.1892	0.0627	6.471
Within 30 days	y = − 1.861x + 20.03	− 3.768 to 0.04726	0.1994	0.0553	7.003
Within 7 days	y = − 2.279x + 22.66	− 5.058 to 0.5013	0.1496	0.1018	6.707
**Average glucose**					
Within 120 days	y = 38.07x − 128.8	34.84 to 41.31	0.9732	< 0.0001	137.69
Within 90 days	y = 37.58x − 125.5	34.03 to 41.13	0.9671	< 0.0001	137.56
Within 60 days	y = 38.40x − 132.5	35.26 to 41.54	0.9751	< 0.0001	136.30
Within 30 days	y = 37.60x − 129.9	32.83 to 42.36	0.9423	< 0.0001	133.30
Within 7 days	y = 36.07x − 117.0	29.99 to 42.14	0.9024	< 0.0001	135.49

*TIR* time in range (glucose level: 70–180 mg/dL), *TAR* time above range (glucose level: > 180 mg/dL), *TBR* time below range (glucose level: < 70 mg/dL), *CI* confidence interval, *R*^*2*^ coefficient of determination.

**Figure 2:**
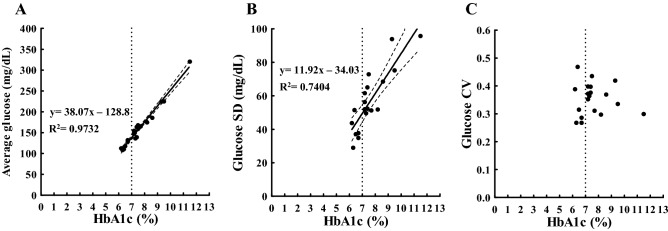
Scatterplot indicating the relationship between HbA1c (x-axis) and CGM-related metrics (average glucose (**A**), glucose SD (**B**), and glucose CV (**C**); y-axis) measured within 120 days from baseline. The solid line is the line of best-fit from the linear regression analysis. The dashed band is the 95% confidence band of the best-fit line. The dotted vertical lines represent an HbA1c of 7%. *SD* standard deviation, *CV* coefficient of variation, *R*^*2*^ coefficient of determination.

**Figure 3:**
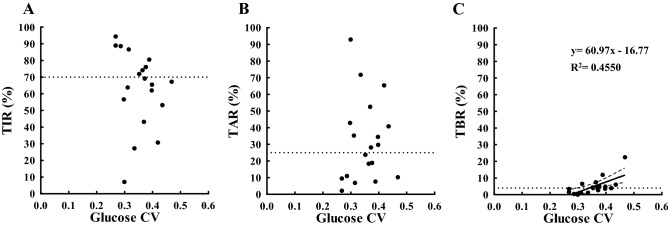
Scatterplot indicating the relationship between glucose CV (x-axis) and CGM-related metrics (TIR (**A**), TAR (**B**), and TBR (**C**); y-axis) measured within 120 days from baseline. The solid line is the line of best-fit from the linear regression analysis. The dashed band is the 95% confidence band of the best-fit line. The dotted horizontal lines represent a TIR of 70% (**A**), a TAR of 25% (**B**), a TBR of 4% (**C**): these values are based on the targets of TIR, TAR, and TBR for adults with type 1 or type 2 diabetes from recommendations of the ATTD 2019 consensus statement. *TIR* time in range (glucose 70–180 mg/dL), *TAR* time above range (glucose ≥ 181 mg/dL), *TBR* time below range (glucose ≤ 69 mg/dL), *R*^*2*^ coefficient of determination.

In Fig. 2A, we observe a strong positive relationship between HbA1c and average glucose (P < 0.0001), suggesting that HbA1c had sufficient data to explain average glucose. HbA1c of 7% corresponded to an average glucose of approximately 138 mg/dL (7.7 mM) (Table 1). A decrease of 39 mg/dL (2.6 mM) in average glucose will cause a decrease in HbA1c of 1.0% (Supplementary Fig. 1C), indicating that HbA1c of 6% of the study population corresponded to an average glucose of approximately 100 mg/dL (5.6 mM). In the remaining CGM-related metrics, glucose SD positively correlated with HbA1c (P < 0.0001) (Fig. 2B), whereas glucose CV and HbA1c were not related (P > 0.05) (Fig. 2C). The mean of glucose CV was 0.35 (range 0.27–0.47). Glucose CV did not correlate with TIR and TAR (P > 0.05 in both) (Fig. 3A,B) as in the case of HbA1c (Fig. 2C). However, glucose CV significantly correlated with TBR, indicating that the increase in glucose CV was associated with increase in hypoglycemia (Fig. 3C). Since 2 of the 19 patients (No. 3 and 10 in Tables 2 and 3) had fewer readings (< 70%) for “within 120 days,” we performed an analysis excluding them, but the results were similar to the analysis with all the patients.

**Table 2:** Patient characteristics.

Participant number	Age	Gender	BMI	Years since diagnosis	Therapy	Total units of insulin injection per day	Total units of insulin injection per day/BW (kg)	HbA1c (%) of baseline	Fasting serum CPR (ng/ml)
1	46.4	Female	18.1	1.3	MDI	20.0	0.43	7.4	0.277
2	74.2	Female	14.9	13.3	MDI	27.0	0.86	9.5	< 0.01
3	61.7	Male	29.7	19.3	CSII	48.2	0.66	9.3	< 0.01
4	70.7	Female	22.0	25.8	CSII	51.6	1.04	7.4	< 0.01
5	34.2	Female	25.0	1.2	CSII	48.0	0.82	6.5	< 0.01
6	70.5	Female	20.6	30.8	MDI	32.0	0.69	6.4	< 0.01
7	70.9	Female	20.3	23.8	MDI	8.0	0.18	6.3	1.21
8	55.2	Female	20.3	0.7	MDI	23.0	0.44	6.7	1.13
9	38.4	Female	23.6	3.8	CSII	25.5	0.39	8.6	< 0.01
10	30.5	Male	27.9	14.8	CSII	50.2	0.63	11.5	< 0.01
11	32.3	Female	21.0	0.6	CSII	32.5	0.76	7.5	< 0.01
12	62.3	Male	19.6	1.4	MDI	33.0	0.53	7.2	0.311
13	39.0	Female	18.9	0.9	CSII	34.0	0.74	6.2	0.409
14	42.6	Male	27.5	9.0	MDI	53.0	0.65	7.2	< 0.01
15	43.2	Male	23.0	3.6	CSII	30.4	0.45	7.7	0.318
16	63.1	Female	16.8	4.0	MDI	17.0	0.42	7.3	0.064
17	53.1	Male	23.2	1.9	MDI	15.0	0.19	6.7	0.931
18	75.8	Female	21.1	5.0	MDI	18.0	0.37	8.2	0.285
19	45.3	Female	19.1	6.3	CSII	26.0	0.55	7.2	0.030
Total or mean ± SD	53.1 ± 15.2	Female: 13, Male: 6	21.7 ± 3.8	8.8 ± 9.6	MDI: 10, CSII: 9	31.2 ± 13.5	0.57 ± 0.22	7.6 ± 1.3	0.261 ± 0.397^a^

*BMI* body mass index, *BW* body weight, *CPR* C-peptide immunoreactivity, *CSII* continuous subcutaneous insulin infusion, *MDI* multiple dose insulin injection.

^a^The values of serum CPR “ < 0.01” were considered as 0 and calculated. Fasting CPR was measured on at least three different dates and the value on the day closest to the baseline date were adopted.

**Table 3:** HbA1c and number of data analyzed.

Participant number	HbA1c (%)					Number of data analysed (% mounting time of CGM in the period^a^)				
	Four months before	Three months before	Two months before	One month before	Baseline	Within 120 days	Within 90 days	Within 60 days	Within 30 days	Within 7 days
1	7.3	7.3	7.2	7.2	7.4	9136 (79.0)	6742 (77.6)	4402 (75.8)	2154 (73.6)	593 (82.4)
2	8.2	8.8	8.9	9.2	9.5	10,939 (94.6)	8209 (94.5)	5537 (95.3)	2786 (95.2)	686 (95.3)
3	9.3	9.8	9.6	9.8	9.3	7621 (65.9)	5234 (60.2)	3276 (56.4)	1648 (56.3)	479 (66.5)
4	7.3	7.6	7.5	7.0	7.4	10,188 (88.1)	7675 (88.3)	5559 (95.7)	2740 (93.6)	692 (96.1)
5	6.8	6.7	6.7	6.5	6.5	10,656 (92.1)	7972 (91.8)	5123 (88.2)	2263 (77.3)	75 (10.4)
6	6.6	6.7	6.7	6.5	6.4	11,399 (98.5)	8548 (98.4)	5701 (98.2)	2849 (97.3)	660 (91.7)
7	6.6	6.4	6.4	6.3	6.3	11,076 (95.7)	8217 (94.6)	5366 (92.4)	2514 (85.9)	700 (97.2)
8	6.4	6.6	6.5	6.5	6.7	10,585 (91.5)	7955 (91.6)	5183 (89.2)	2408 (82.2)	462 (64.2)
9	8.4	8.4	8.3	8.4	8.6	10,852 (93.8)	7997 (92.0)	5162 (88.9)	2303 (78.7)	130 (18.1)
10	11.8	11.8	12.1	12.2	11.5	5376 (46.5)	4260 (49.0)	3517 (60.6)	2649 (90.5)	669 (92.9)
11	7.4	7.0	7.7	7.9	7.5	11,316 (97.8)	8479 (97.6)	5654 (97.3)	2809 (95.9)	704 (97.8)
12	6.9	6.9	7.3	7.4	7.2	11,464 (99.1)	8610 (99.1)	5759 (99.2)	2898 (99.0)	713 (99.0)
13	6.1	6.0	5.9	6.2	6.2	9769 (84.4)	7224 (83.1)	5023 (86.5)	2800 (95.6)	693 (96.3)
14	6.9	7.3	7.3	7.1	7.2	9650 (83.4)	6921 (79.7)	5416 (93.3)	2783 (95.0)	690 (95.8)
15	7.1	6.9	7.0	7.4	7.7	10,052 (86.9)	7507 (86.4)	5090 (87.6)	2558 (87.4)	701 (97.4)
16	7.0	7.0	7.1	7.4	7.3	11,499 (99.4)	8634 (99.4)	5780 (99.5)	2912 (99.5)	707 (98.2)
17	6.7	6.5	6.7	6.5	6.7	11,337 (98.0)	8502 (97.9)	5664 (97.5)	2822 (96.4)	694 (96.4)
18	8.0	8.4	8.2	8.4	8.2	11,384 (98.4)	8563 (98.6)	5737 (98.8)	2899 (99.0)	711 (98.8)
19	7.0	6.9	7.2	7.1	7.2	9980 (86.3)	7954 (91.6)	5428 (93.5)	2906 (99.2)	724 (100.6)
Mean ± SD	7.5 ± 1.3	7.5 ± 1.4	7.6 ± 1.4	7.7 ± 1.5	7.6 ± 1.3	10,225 ± 1534 (88.4 ± 13.3)	7642 ± 1176 (88.0 ± 13.5)	5178 ± 715 (89.2 ± 12.3)	2616 ± 334 (89.3 ± 11.4)	604 ± 192 (84.0 ± 26.6)

*CGM* continuous glucose monitoring.

^a^The number of analyzed data were divided by 11,568, 8688, 5808, 2928 corresponding to maximum points for 120.5, 90.5, 60.5, and 30.5 days, respectively, and shown as % mounting time of CGM.

**Figure 4:**
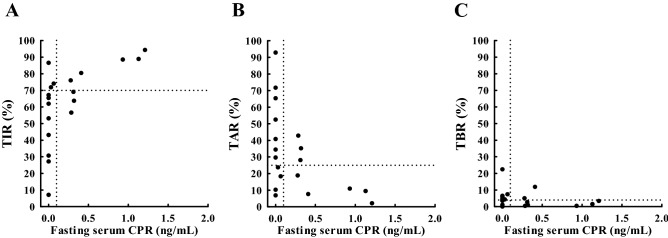
Scatterplot indicating the relationship between fasting serum CPR (x-axis) and CGM-related metrics (TIR (**A**), TAR (**B**), and TBR (**C**); y-axis) measured within 120 days from baseline. The dotted horizontal lines represent a TIR of 70% (**A**), a TAR of 25% (**B**), and a TBR of 4% (**C**). The dotted vertical line represents a fasting serum CPR of 0.1 ng/mL. *TIR* time in range (glucose 70–180 mg/dL), *TAR* time above range (glucose ≥ 181 mg/dL), *TBR* time below range (glucose ≤ 69 mg/dL).

As previous studies have indicated serum CPR levels as an essential contributor to glucose variability^15–17^, the relationship between serum CPR and CGM-related metrics was investigated. Wide variations in TIR and TAR were observed in decreased fasting serum CPR (Fig. 4A,B). Markedly low TIR (Fig. 4A), high TAR (Fig. 4B) and high TBR (Fig. 4C) were observed in patients with fasting serum CPR < 0.1 ng/mL.

**Figure 5:**
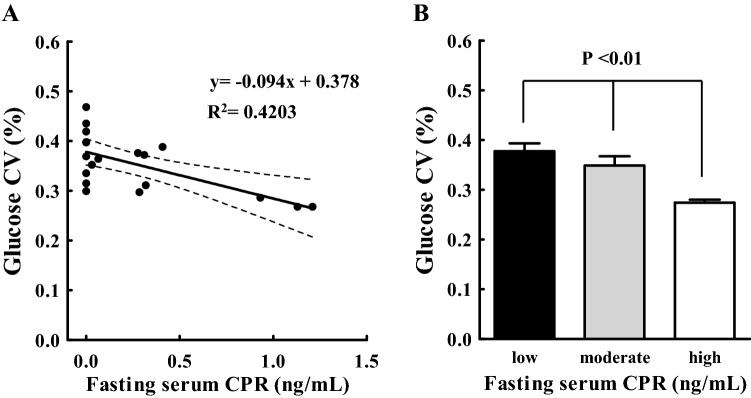
(**A**) Scatterplot indicating the relationship between fasting serum CPR (x-axis) and glucose CV (y-axis) measured within 120 days from baseline. The solid line is the line of best fit from linear regression analysis. The dashed band is the 95% confidence band of the line of best fit. (**B**) Fasting serum CPR levels are divided into three groups: < 0.1 ng/mL (low), > 0.1 ng/mL and < 0.6 ng/mL (moderate), and > 0.6 ng/mL (high). Glucose CV was measured within 120 days from baseline. Glucose CV among different levels of CPR groups was tested using the Jonckheere–Terpstra trend test. A trend toward lower glucose CV with higher serum CPR (p < 0.01) is noted. *CPR* C-peptide, *CV* coefficient of variation, *R*^*2*^ coefficient of determination.

In Fig. 5A, a significant inverse relationship was observed between fasting serum CPR and glucose CV (P < 0.005). An increase in fasting serum CPR was associated with a decrease in glucose CV (P < 0.01, Jonckheere–Terpstra trend test) (Fig. 5B).

In addition to the analysis of CGM data “within 120 days” from baseline described above, we further studied multiple time period measurements from baseline: within 90, 60, 30, and 7 days (Table 1). The results obtained were essentially the same as in the case of “within 120 days.” TIRs measured within 90 and 60 days from baseline showed strong relationship with HbA1c (coefficient of determination (R^2^) = 0.8771 and R^2^ = 0.8851, respectively) as in the case of within 120 days (R^2^ = 0.8881); however, TIRs measured within 30 and 7 days showed a slightly weaker correlation with HbA1c (R^2^ = 0.8489 and R^2^ = 0.7110, respectively). TARs and average glucose showed same tendency as TIRs, namely, a strong relationship with HbA1c for longer periods and slightly weaker for shorter periods. As for TBRs, no significant relationship with HbA1c for any period was observed. Glucose CVs measured within 90, 60, and 30 days from baseline showed strong relationship with fasting serum CPR (R^2^ = 0.4092, R^2^ = 0.3333, and R^2^ = 0.2250, respectively) as in the case of “within 120 days” (R^2^ = 0.4203); however, glucose CVs measured within 7 days showed no correlation with fasting serum CPR (R^2^ = 0.1405).

## Discussion

The present study was performed to clarify the advantages and disadvantages of CGM-related metrics relative to well-established laboratory-measured parameters, HbA1c and serum CPR, in type 1 diabetes. TIR, TAR, and average glucose levels, but not TBR, strongly correlated with HbA1c. TBR correlated with glucose CV. Fasting serum CPR was negatively correlated with glucose CV. HbA1c of approximately 7% corresponded to TIR of 74% and TAR of 20%. The shorter the CGM period, the weaker the relationship between HbA1c and CGM-related metrics. To date, numerous studies have been published on the relationship between CGM-related metrics and HbA1c^11,13^. However, most studies have been based on data extracted from published trials or articles for other purposes and does not exist as studies specified in measurement terms, to investigate for CGM data for a sufficiently long period of time^11,13,18^. To exactly investigate the relationship between HbA1c and CGM-related metrics, the measurement periods were fixed as 120, 90, 60, 30, and 7 days from the baseline. To avoid discrepancy due to CGM-related metrics reflecting short-term glycemic status and HbA1c reflecting long-term glycemic status, only those patients with stable glycemic control and minimal changes in HbA1c four months prior to the baseline were considered for analysis, thus resulting in less than 15% variation in HbA1c. Despite a relatively small number of patients, a very strong relationship between CGM-related metrics, TIR, TAR, and particularly average glucose levels, and HbA1c was detected, indicating that CGM-related metrics TIR and TAR could adequately reflect laboratory-measured HbA1c provided that HbA1c levels have been stable within the past several months.

ATTD has indicated three key CGM-related metrics: TIR, TAR, and TBR^2^. The ATTD report has described that CGM-based glycemic targets must be personalized to meet the needs of each person with diabetes. In addition, the report had proposed a glycemic range (a target range of 70–180 mg/dL) and targets of TIR, TAR, and TBR for adults with type 1 or type 2 diabetes (TIR > 70%, TAR < 25%, TBR < 4%) and older/high-risk individuals (TIR > 50%, TAR < 50%, TBR < 1%). Two studies have reported that TIR of 70% and 50% corresponded to an HbA1c of approximately 7% and 8%^11^, and 6.7% and 8.3%^13^, respectively. However, the present study on adult Japanese patients with type 1 diabetes showed that TIRs of 70% and 50% corresponded to HbA1c of approximately 7.3% and 8.4%, respectively (best-fit line: y = − 0.05412x + 11.06; when x = TIR, y = HbA1c) (Supplementary Fig. 1A). To achieve HbA1c of 7% and 8%, TIR should be more than 74% and 57%, respectively. TAR matched more with HbA1c compared to TIR or TBR, in our population. TAR of 25% and 50% corresponded with HbA1c of approximately 7.3% and 8.6%, respectively (best-fit line: y = 0.05211x + 5.969; when x = TAR, y = HbA1c) (Supplementary Fig. 1B). To achieve HbA1c of 7% and 8%, TAR should be less than 20% and 39%, respectively.

TBR showed only marginal correlation with HbA1c. This may be due to a limited TBR compared to TIR and TAR. However, a tendency of reducing HbA1c with increasing TBR suggests the limitation of HbA1c and the advantage of the differentiation to increase TIR and simultaneously decrease or at least have unchanged TBR. In addition, as shown in Fig. 3C, a significant relationship between glucose CV and TBR was observed; therefore, higher the glucose CV, higher was TBR (risk of hypoglycemia). These results demonstrated the need to maintain glucose CV below a certain level (e.g., < 0.36^19^) to avoid hypoglycemia in patients at an increased risk. However, it is often difficult to regulate glucose CV in type 1 diabetes, particularly, in the subset of patients with unstable (brittle) diabetes. Even in patients with stable HbA1c in our study, the mean glucose CV was 0.35. One major factor contributing to glucose CV is residual beta-cell function^15–17^. Wide variations in TIR and TAR in patients with low CPR (Fig. 4A,B) together with the inverse relationship between glucose CV and serum CPR (Fig. 5A,B) indicate the importance of residual β-cell function on glycemic control stabilization. Our data regarding CGM-derived glucose CV are consistent with those of previous reports on the negative correlation between serum CPR and glucose variability assessed in pre-CGM era^15–17^. It emphasizes the importance of residual CPR, which is reported to be associated with the progression of diabetes complications^20^, on glycemic stability in type 1 diabetes. Most Japanese patients with type 1 diabetes were reported to completely lose endogenous insulin during the disease duration^21^; inversely most patients with long-duration type 1 diabetes in Western countries preserved endogenous insulin levels^22,23^, and treatment to prevent CPR decline has been reported to inhibit the progression of diabetes complications in patients with residual CPR^24^. Therefore, CGM-derived TBR and glucose CV together with CPR are essential metrics supplementing HbA1c in patients with type 1 diabetes, particularly in the Japanese population.

Average glucose, rather than other CGM-related metrics, exactly matched with HbA1c, particularly, in the CGM data collected from the last 60 days or more. CGM data collected “within 7 days” showed a significant but weaker relationship with HbA1c and average glucose than those from longer periods (Table 1). These results confirmed that HbA1c is an index for long-term monitoring of blood glucose and reflects mean glucose levels during the last 2 months, as reported previously^1,2^. This study was designed to use CGM data only in patients with stable glycemic control with minimal changes in HbA1c during the 4 months before the baseline. Even then, a duration dependency was observed regarding the correlation between CGM-related metrics and HbA1c, shorter duration of CGM-derived data, weaker was the correlation with HbA1c. The correlation would be even weaker in case CGM-derived data are extracted from clinical trials or articles in which glycemic control changes due to interventions or treatments resulting in CGM-derived data reflecting short-term glycemic control changes much faster than HbA1c. In fact, only a moderate correlation was reported between CGM-related metrics and HbA1c with a correlation coefficient of 0.67–0.73 for TIR in 545 patients with type 1 diabetes from 4 randomized trials^11^. From this point of view, previous studies on the relationship between HbA1c and CGM-related metrics and estimation of HbA1c from CGM-related metrics using short-term CGM data should be carefully interpreted.

Petersson et al. reported the relationship between HbA1c and CGM-related metrics, focusing on time in target range (TIT) defined as 70–140 mg/dL^25^; this is the first report to identify a nonlinear relationship between TIT and HbA1c in pediatric type 1 diabetes. However, in the same paper, there is a description of a linear analysis of TIT 30, 60, and 90 days and HbA1c. All of them showed significant correlations, but the correlation coefficients (R^2^: 0.59–0.63) were lower than R^2^ for TIR in the present study. These differences may be due to the difference in race (Japanese vs. Swedish), subjects (adults vs. children and adolescents), selection criteria (stable HbA1c population vs. general population), and CGM-metrics (TIR vs. TIT). The relationship between HbA1c and CGM-metrics relative to CGM duration, which is our main objective in the present study, is unknown because the relationship in shorter (7 days) and longer (120 days) CGM duration is not available in their study.

The main strength of this study is that it was specifically designed to test the correlation between CGM-related metrics and HbA1c; it evaluated the accuracy of CGM-related metrics for different CGM measurement periods. Moreover, this is the first report on adult Japanese patients with type 1 diabetes, a population prone to insulin depletion.

This study had limitations. First, only a limited number of patients were eligible for investigation because we considered a study design for data collection to increase the accuracy of the results; herein, we selected for each patient the period with stable glycemic control with small changes in HbA1c. In addition, we excluded patients with clinical conditions unrelated to glycemia but affecting HbA1c levels, such as unstable blood hemoglobin values, gestational period, surgery, and diagnosis of malignant tumors. Due to the exclusion of subjects with these conditions, the results of the present study may not apply to patients with type 1 diabetes in general. Second, the relationship between HbA1c and average glucose was studied only with respect to CGM-derived glucose value, but not with laboratory-measured plasma glucose, as our study was performed under daily life conditions. Considering the recent developments in CGM technology with improvement in sensor accuracy, the data obtained with CGM for real-life glucose in normal daily use provide information difficult to be obtained with the laboratory-measured glucose, under restricted settings such as hospitals. Third, as our patients use FreeStyle Libre, which has dual function, CGM and FGM, there may be biases based on patients’ habits. For example, when unexpected high or low glucose values were detected using FGM, patients willingly compensated their blood glucose, leading to better than the expected TIR levels. Fourth, the analysis of the relationship between glucose CV and fasting serum CPR was performed using only univariate regression analysis because of the limited number of the subjects in this study. For this reason, the observed relationship can be indirect and secondary to other unknown factors. Although our data on CGM-derived glucose CV are consistent with those of previous reports of glucose variability estimated using different methods in pre-CGM era^15–17^, further studies with larger number of participants are required to clarify the observed relationship with the consideration for other confounding factors. Fifth, although the relationship between glucose CV and TBR was detected in this study, the relationship may be overrated or underrated. This is because residual CPR may potentially affect both glucose CV and TBR. Additionally, Japanese patients with type 1 diabetes are known to be prone to CPR depletion.

In conclusion, this study provided evidence regarding the reliability of CGM-related metrics in adult Japanese patients with type 1 diabetes in a usual daily life setting. TIR and TAR strongly correlated with HbA1c and complemented or even replaced HbA1c for short-term monitoring of blood glucose. While TIR, TAR, and HbA1c reflected mean blood glucose, TBR and glucose CV complemented the mean glucose-related metrics by reflecting hypoglycemia. The negative correlation between glucose CV and fasting CPR suggests the importance of residual β-cell function for stable glycemic control without hypoglycemia, in type 1 diabetes.

## Methods

### Patients

Nineteen patients with type 1 diabetes who regularly visited the Kindai University Faculty of Medicine (Osaka-sayama, Osaka, Japan) once a month for more than 5 months were included in the study, between December 2018 and December 2019. Patient characteristics are given in Table 2. All patients were adults (age range 30.5–75.8, mean ± SD 53.1 ± 15.2) with type 1 diabetes receiving multiple insulin injections or continuous subcutaneous insulin infusion. The protocols were approved by the Ethics Review Committee of the Kindai University Faculty of Medicine (approval number: 31-162), and informed consent was obtained from all participants. All methods were performed in accordance with the relevant guidelines and regulations.

### Study design

Patients’ data were collected in their daily life settings. The patients at their visit to our hospital, brought with them their FreeStyle Libre device (Abbott Japan Diabetes Care Inc., Tokyo, Japan), which is a flash glucose monitoring (FGM) system that has dual function, CGM and FGM. The FreeStyle Libre data were extracted and stored in a computer solely for this purpose. CGM measures every 15 min the interstitial fluid glucose and converts it to venous blood glucose; however, in FGM, glucose data are not constantly shown and are available only on demand. In this study, only CGM data were used for analysis.

Patients who met all of the following five criteria were included in the study: (1) stable HbA1c, which was defined as minimum difference in HbA1c levels for the past 4 months in each patient, and stable blood hemoglobin levels in the past 4 months, (2) no gestational period, (3) no operations within the last 6 months and no planned operations, (4) no diagnosis for malignant tumors, (5) no clinical conditions unrelated to glycemia but affecting HbA1c levels, such as anemia, renal failure and liver cirrhosis. HbA1c at baseline and at 1 to 4 months before baseline is shown in Table 3. In our institution, about 200 patients with type 1 diabetes are provided treatment through regular outpatient clinic visits, and 26 patients used Freestyle Libre during the period of this study. Of these, 7 patients were excluded because of the following reasons: CGM data of less than 4 months were available, blood data were not available, and/or inclusion criteria were not met. Eventually, 19 patients were included in the analysis.

The CGM-related metrics were calculated, namely TIR (glucose 70–180 mg/dL), TAR (glucose ≥ 181 mg/dL), TBR (glucose ≤ 69 mg/dL), average glucose, glucose standard deviation (SD), and glucose CV for each patient using the CGM data within 120 days from baseline. Overall, 194,279 CGM values (average 10,225 per patient) were analyzed. In addition, we calculated CGM-related metrics based on CGM data closer to the baseline, “within 90 days,” “within 60 days,” “within 30 days,” and “within 7 days” from baseline. The number of data analyzed and the percentage mounting time of CGM in the corresponding period are provided in Table 3. On an average, the percentage mounting time of CGM was over 84%.

In addition to the relationship between CGM-related metrics and HbA1c, the relationship between CGM-related metrics and serum CPR was analyzed to study the effect of residual β-cell function on glycemic control, particularly fluctuation (glucose CV) and hypoglycemia (TBR).

### Statistical analysis

Univariate linear regression was used to characterize the relationship between HbA1c and CGM-related metrics, and between fasting serum CPR and glucose CV. The relationship between the trend of fasting serum CPR improvement and glucose CV was analyzed using the Jonckheere–Terpstra trend test. Statistical tests were performed using GraphPad Prism (GraphPad software) or Bell Curve for Excel (Social Survey Research Information Co., Ltd.). P < 0.05 was considered statistically significant.

## Supplementary Information

Supplementary Legend.

Supplementary Figure.
