# Supplementary material for: Relationship of continuous glucose monitoring-related metrics with HbA1c and residual β-cell function in Japanese patients with type 1 diabetes
Source: Sci Rep. 2021 Feb 17;11:4006. doi: 10.1038/s41598-021-83599-x (PMC7889608; doi:10.1038/s41598-021-83599-x)
Supplement: Supplementary file 1 — Supplementary Legend. [file 41598_2021_83599_MOESM1_ESM.docx]

**Supplement Figure Legends**

**Supplement Figure 1.** Scatterplot indicating the relationship between HbA1c (y-axis) and CGM-related metrics (TIR (A), TAR (B), and average glucose (C); y-axis) measured within 120 days from baseline. In these figures, the x- and y-axis of Figure 1A, 1B and Figure 2A are simply swapped. The solid line is the line of best fit from the linear regression analysis. The dashed band is the 95% confidence band of the best-fit line. The dotted horizontal lines represent an HbA1c of 7%. The dotted vertical lines represent a TIR of 70% (A), a TAR of 25% (B): these values are based on the targets of TIR and TAR for adults with type 1 or type 2 diabetes from recommendation of the ATTD 2019 consensus statement. TIR: time in range (glucose 70-180 mg/dL), TAR: time above range (glucose ≥181 mg/dL), R^2^: coefficient of determination
