# Supplementary material for: Relationship of continuous glucose monitoring-related metrics with HbA1c and residual β-cell function in Japanese patients with type 1 diabetes
Source: Sci Rep. 2021 Feb 17;11:4006. doi: 10.1038/s41598-021-83599-x (PMC7889608; doi:10.1038/s41598-021-83599-x)
Supplement: Supplementary file 2 — Supplementary Figure. [file 41598_2021_83599_MOESM2_ESM.pptx]

## Slide 1
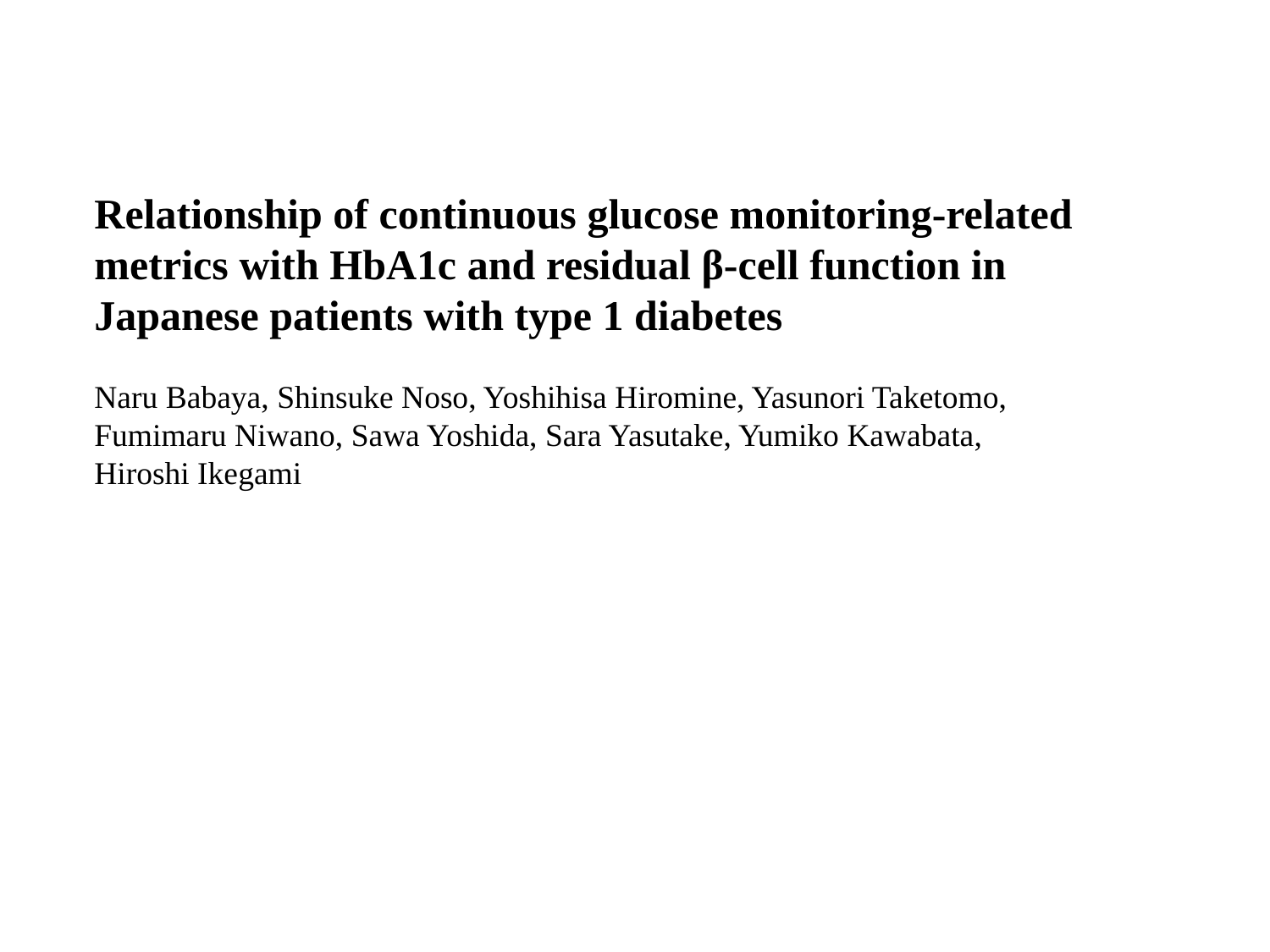

Relationship of continuous glucose monitoring-related metrics with HbA1c and residual β-cell function in Japanese patients with type 1 diabetes
Naru Babaya, Shinsuke Noso, Yoshihisa Hiromine, Yasunori Taketomo,
Fumimaru Niwano, Sawa Yoshida, Sara Yasutake, Yumiko Kawabata,
Hiroshi Ikegami

## Slide 2
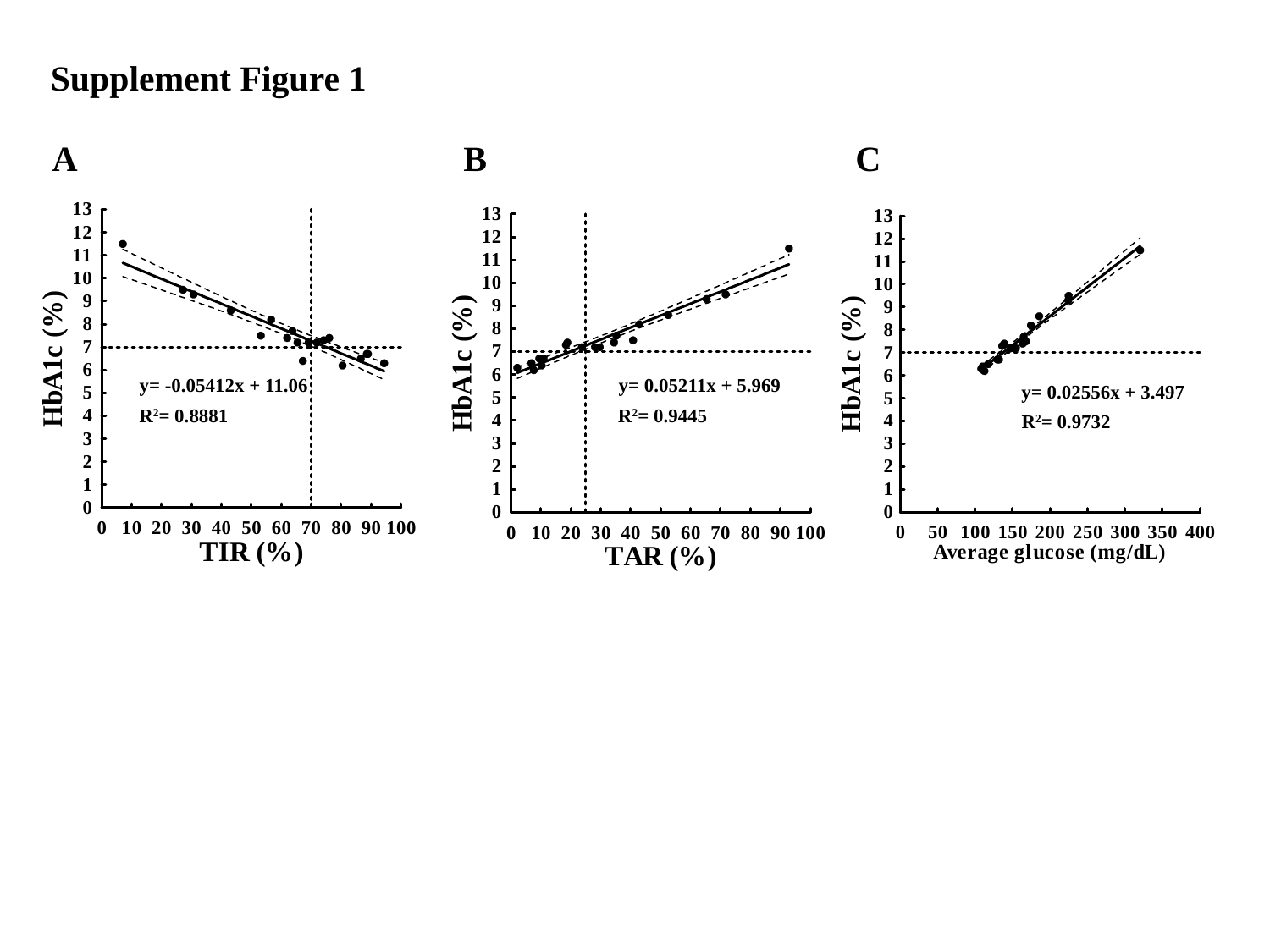

Supplement Figure 1
A
B
C
y= -0.05412x + 11.06
y= 0.05211x + 5.969
y= 0.02556x + 3.497
R2= 0.8881
R2= 0.9445
R2= 0.9732
